# Comment on ‘Systematic Review and Meta‐Analysis of Protein Intake to Support Muscle Mass and Function in Healthy Adults’ by Nunes et al.—The Authors' Reply

**DOI:** 10.1002/jcsm.70108

**Published:** 2025-10-22

**Authors:** Everson A. Nunes, Daniel Tomé, Sandra Naranjo‐Modad, Diana Sherifali, Claire Gaudichon, Stuart M. Phillips, the authors

**Affiliations:** ^1^ Department of Human Health Sciences University of Guelph Guelph Ontario Canada; ^2^ Department of Kinesiology, Protein Metabolism Research Laboratory McMaster University Hamilton Ontario Canada; ^3^ Université Paris‐Saclay, AgroParisTech, INRAE UMR PNCA Paris France; ^4^ Givaudan France Naturals, Taste and Wellbeing Avignon France; ^5^ School of Nursing, Faculty of Health Sciences McMaster University Hamilton Ontario Canada

Dear Editor,

1

We read the letter by Inca‐Barriga et al. [1] regarding our systematic review and meta‐analysis published in 2022 [2]. The concerns these authors raise essentially misinterpret our methodology, miss key details clearly described in our paper, and fail to recognize the robustness of our analytical approach.

First, regarding the calculation of effect sizes. It was suggested that our use of change scores could inflate effect estimates. This assertion reflects a misunderstanding of our methods. As stated in both the Cochrane Handbook (v6.5 [2024], Sections 10.5.2 and 6.5.1) [3] and widely accepted meta‐analytic practice, effect sizes may be derived from either post‐intervention values or change‐from‐baseline values, provided the calculation is performed appropriately. Our analysis did not rely solely on within‐group pre–post changes from intervention groups, as incorrectly implied by the critique. Instead, we employed the difference‐in‐differences methodology: for each study, we computed the mean change from baseline for both intervention and control groups, followed by the calculation of the between‐group difference, standardized by the pooled standard deviation. This approach ensures that the between‐group comparison—the fundamental element of causal inference in randomized trials—remains intact and preserved. Far from inflating effect sizes, this approach prevents the type of statistical error highlighted by Cuijpers et al. [4], where control data are disregarded. (As an example of meta‐analyses excluding control data from the effect size calculation, please see [5, 6, 7].) Thus, our method is not only fully consistent with Cochrane guidance but also represents the most rigorous way to estimate intervention effects.

Second, concerns were raised regarding potential unit‐of‐analysis errors arising from the inclusion of multiple arms from the same study. This issue was explicitly anticipated and directly addressed in our methodology. As we clearly reported (Methods, p. 798) [2], we employed a three‐level random‐effects meta‐analytic model, which decomposes variance into sampling, within‐study, and between‐study components. This statistical framework is specifically designed to handle dependencies across multiple effect sizes from the same trial, thereby avoiding any inflation of precision or violation of independence assumptions. Suggesting otherwise disregards the methodological sophistication of our approach and the transparency with which it was reported.

Concerns were also raised about the inclusion of certain studies in Figure 2. All studies included in our analysis were screened against predefined PICOS criteria (Table 1) [2], and their inclusion was based on methodological fit, not statistical convenience. The Burke et al. study [8], for example, met these criteria. Nevertheless, excluding this study, as suggested, produces virtually identical effect estimates (0.19 [0.11, 0.28] vs. 0.19 [0.10, 0.27]), underscoring that the findings are not contingent upon its inclusion. Similarly, sensitivity analyses already conducted and reported in our original manuscript demonstrated that excluding Willoughby et al. [9] or Nakayama et al. [10] had negligible or expected subgroup effects. Indeed, we explicitly acknowledged that removing Nakayama et al. [10] changed one subgroup estimate to non‐significance—a limitation we reported transparently (p. 802) [2].

To further test the robustness of our conclusions, we conducted additional analyses, simultaneously excluding Burke et al. [8], Willoughby et al. [9], Nakayama et al. [10], and Oertzen‐Hagemann et al. [11]. The overall effect size remained statistically significant (original: 0.19 [0.11, 0.28] vs. excluding all four studies: 0.15 [0.06, 0.23]), as did the ≥ 1.6 g/kg/day subgroup estimate (original: 0.30 [0.17, 0.43] vs. excluding: 0.23 [0.10, 0.37]). These findings confirm that our conclusions are robust, consistent, and not driven by any single study or small subset of studies.

The authors reference Helms et al. [12] to argue that energy availability, rather than protein intake, is a main driver of lean mass gains. Yet their own citation contradicts this claim. Helms et al. [12] explicitly concluded that larger energy surpluses increased fat mass, not lean mass, and did not demonstrate superior effects of higher energy availability on muscle accretion. From Helms et al. [12], ‘Therefore, we conclude faster rates of BM [body mass] gain (and by proxy larger surpluses) primarily increase rates of fat gain rather than augmenting 1‐RM [one‐repetition strength] or MT [muscle thickness]’. Citing this paper as evidence against our findings reflects a basic and unfortunate misinterpretation of the premise that the authors claim is affecting our data.

Finally, the assertion that our language implies direct causality is unfounded. While meta‐analyses of randomized controlled trials are recognized as among the highest levels of evidence for causal inference, we deliberately employed measured, conditional, and qualified language throughout. Some examples: In the abstract, we wrote that protein intake ‘may enhance’ lean body mass gains (p. 795) [2]; in the results, we noted that additional intake ‘probably leads to a small increase’ (p. 801) [2]; and in the conclusion, we stated that evidence ‘supports the hypothesis’ (p. 806) [2]. Moreover, we explicitly downgraded the certainty of some findings using GRADE, underscoring the modest effect sizes and the need for caution in interpretation. Such phrasing is the opposite of overstated; it is balanced, transparent, and scientifically rigorous.

In conclusion, the criticisms provided by Inca‐Barriga et al. [1] do not weaken the validity or credibility of our analysis. On the contrary, we have demonstrated that our methodology is firmly grounded in Cochrane‐endorsed practices, our modeling appropriately addresses complex study designs, our findings are robust across multiple sensitivity analyses, and our conclusions are cautiously worded in accordance with the strength of the evidence. Consequently, we find little‐to‐no merit in the arguments presented and reaffirm the robustness, transparency, and scientific integrity of our study.

## Conflicts of Interest

Stuart M. Phillips received funding from several organizations, including the Canadian Institutes of Health Research, the Natural Sciences and Engineering Research Council of Canada, the US National Institutes of Health, Roquette Frères, Nestlé Health Science, FrieslandCampina, the US National Dairy Council, Dairy Farmers of Canada, and Myos. He has also received travel expenses and honoraria from Nestlé Health Science. He holds patents licensed to Exerkine Inc. but reports no financial gains. Sandra Naranjo‐Modad is an employee of Givaudan. Everson A. Nunes reports providing independent scientific consulting in nutrition and metabolism and receiving honoraria and travel support for invited industry‐sponsored lectures. Diana Sherifali, Claire Gaudichon, and Daniel Tomé declare that they have no conflicts of interest.
